# Effects of non-compulsory and mandatory COVID-19 interventions on travel distance and time away from home, Norway, 2021

**DOI:** 10.2807/1560-7917.ES.2023.28.17.2200382

**Published:** 2023-04-27

**Authors:** Meghana Kamineni, Kenth Engø-Monsen, Jørgen E Midtbø, Frode Forland, Birgitte Freiesleben de Blasio, Arnoldo Frigessi, Solveig Engebretsen

**Affiliations:** 1 Oslo Centre for Biostatistics and Epidemiology, University of Oslo and Oslo University Hospital, Oslo, Norway; 2 Telenor Research, Oslo, Norway; 3 Department of Method Development and Analytics, Norwegian Institute of Public Health, Oslo, Norway; 4 Division of Infection Control, Norwegian Institute of Public Health, Oslo, Norway; 5 Norwegian Computing Center, Oslo, Norway

**Keywords:** SARS-CoV-2, Non-pharmaceutical interventions, mobile phone mobility, adherence to advice, adherence to mandates, synthetic difference-in-differences

## Abstract

**Background:**

Given the societal, economic and health costs of COVID-19 non-pharmaceutical interventions (NPI), it is important to assess their effects. Human mobility serves as a surrogate measure for human contacts and compliance with NPI. In Nordic countries, NPI have mostly been advised and sometimes made mandatory. It is unclear if making NPI mandatory further reduced mobility.

**Aim:**

We investigated the effect of non-compulsory and follow-up mandatory measures in major cities and rural regions on human mobility in Norway. We identified NPI categories that most affected mobility.

**Methods:**

We used mobile phone mobility data from the largest Norwegian operator. We analysed non-compulsory and mandatory measures with before–after and synthetic difference-in-differences approaches. By regression, we investigated the impact of different NPI on mobility.

**Results:**

Nationally and in less populated regions, time travelled, but not distance, decreased after follow-up mandatory measures. In urban areas, however, distance decreased after follow-up mandates, and the reduction exceeded the decrease after initial non-compulsory measures. Stricter metre rules, gyms reopening, and restaurants and shops reopening were significantly associated with changes in mobility.

**Conclusion:**

Overall, distance travelled from home decreased after non-compulsory measures, and in urban areas, distance further decreased after follow-up mandates. Time travelled reduced more after mandates than after non-compulsory measures for all regions and interventions. Stricter distancing and reopening of gyms, restaurants and shops were associated with changes in mobility.

**Key public health message**

**What did you want to address in this study?**

COVID-19 restrictions have many costs for society, including financial costs and effects on citizens’ mental and physical health. We wanted to understand how compliant people were with non-mandatory and follow-up mandatory measures in Norway, which we measured through their impact on reducing human mobility. If non-mandatory measures are effective in reducing mobility, they may be sufficient to generate compliance.

**What have we learnt from this study?**

People travelled shorter distances from home after non-compulsory measures were introduced. In urban areas, distance decreased further when the measures were made mandatory. Travelling times also became shorter, and this effect was stronger after mandated than after non-compulsory interventions, in both rural and urban regions. Stricter metre rules and reopening of gyms, restaurants and shops prompted changes in people’s behaviour.

**What are the implications of your findings for public health?**

In less populated areas, making the recommendations mandatory did not make people reduce their travelling distance much more than they already had after the initial recommendations. Therefore, less invasive and costly non-mandatory measures may be sufficiently effective in less urbanised areas in Norway.

## Introduction

Current understanding of the effects of individual non-pharmaceutical interventions (NPI) on COVID-19 transmission is still limited [1]. We studied the effects of NPI on human mobility, serving as a proxy for compliance and contacts beyond households [2].

It is difficult to analyse how NPI impact infections in observational studies owing to seasonality, new strains, self-regulating behaviour and multiple interventions being implemented and/or lifted simultaneously. Moreover, interventions are often lifted during periods with little transmission and few cases, so finding significant differences requires strong effects. Few randomised clinical trials for COVID-19 interventions exist for ethical, political, and logistical reasons, leading to a lack of evidence [3].

Because of these limitations with analysing infections, some studies have investigated how COVID-19 NPI affect mobility [4,5]. One study of 56 countries concluded that lockdowns and states of emergency reduced mobility [6]. Other studies have analysed the effects of social distancing interventions on mobility in American socio-economic groups [7] and on geographical regions in the United Kingdom (UK) [8].

While many countries have implemented mandatory NPI to curb COVID-19 transmission, Norway, alongside the other Nordic countries, has extensively employed non-mandatory advice to reduce social contacts and sometimes, during epidemic spikes, made these interventions obligatory [9]. Non-mandatory measures are less invasive and costly than stricter alternatives and have been recommended in previous pandemics, including influenza [10-12]. However, there is limited knowledge about the effects of COVID-19 non-mandatory NPI, although one study from Tokyo found that recommendations led to reduced mobility [13]. Since non-mandatory measures were often made mandatory in Norway, we can compare the impact that adding mandatory interventions vs keeping non-mandatory interventions has on mobility. If follow-up mandates of recommendations did not result in further reductions, we can conclude that mandates did not provide significant additional compliance. We also analysed the effects of specific NPI, such as limitations on private guests, events and alcohol serving.

Investigating the effects of NPI on mobility requires careful choice of data, and we used mobile phone data that are unique in their high coverage of the Norwegian population, estimated at ca 47.5% of mobile phone users in 2019 [14]. Many studies have used data that capture overall movements of individuals relative to baselines such as Google data [15]. We propose alternate metrics, radius of gyration and time away from home, which provide different insights into human behaviour compared with origin–destination mobility data. Radius of gyration describes distance travelled from home and is useful to study regional interventions thanks to its sensitivity to local effects [16]. It has been used to predict COVID-19 deaths and to analyse travel restrictions in Austria, Italy, Thailand and Tokyo [13,16-19]. A study from the United States (US) analysing stay-at-home orders explored time spent at home and found that urban populations travelled less than rural populations [20]. We aimed to use these alternate mobility metrics on a large representative dataset to better understand the effects of NPI on mobility.

## Methods

### Data and pre-processing

We compiled national and local COVID-19 NPI from governmental websites and news sources. Individual sources for interventions are listed in Supplementary Table S1. We considered an NPI mandatory if citizens are obliged to follow it by regulation or law. As a non-mandatory NPI we considered a recommendation that citizens are advised to follow, but non-compliance would not have legal consequences. Analysis of local interventions focused on the following major cities: Oslo, Stavanger, Tromsø, Trondheim and Bergen (see Supplementary Figure S1 for a map of Norway). We excluded interventions related to Norwegian border crossings, e.g. travel bans and quarantine requirements. We included interventions with regulations on the following: teleworking, face mask use, private guest limits, physical distancing, alcohol serving, events, schools, gyms, restaurants, shops and businesses. We excluded interventions with fewer than 5 days of mobility data in the week before or after.

We used mobile phone data from the largest Norwegian operator, Telenor, from 24 January 2021 to 9 January 2022. Each phone is connected to a cell tower, typically the closest location. By tracking phones connected to towers, we can follow movements of devices. We collected three mobility metrics per day per individual, aggregated into distributions for each of the 356 municipalities in Norway. The three metrics are radius of gyration time weighted (meanDistAway) which measures distance travelled from home (the location of the individual at 4:00 h), time spent away from home (timeAway), and maximum distance travelled away from home (maxDistAway) (Table 1); In Supplement section A, we provide more details on the derivation of these metrics. To increase privacy protection and ensure that individuals cannot be traced by their location history, data are unavailable every third Monday, as the data provider regularly changed the hashing/pseudonymisation keys of users.

**Table 1:** The three measures of individually measured daily mobility used in this study on COVID-19 interventions and mobility, Norway, 2021

Mobility metrics	Abbreviated name	Definition
Radius of gyration	meanDistAway	Mean cumulative distance travelled from home during the day, weighted by time spent at each location.
Time away from home	timeAway	Amount of time during the day a subscriber spends connected to a cell tower other than their home tower.
Max distance away from home	maxDistAway	Maximum Euclidean distance between the home tower and all other towers to which the subscriber is connected during the day.

A day starts at 4:00 h and finishes 24 h later. Precise definitions and derivations of all metrics are provided in the Supplement, section A.

We determined daily mean meanDistAway, timeAway, maxDistAway on national, county and municipality levels. We used these metrics to compare national non-mandatory and follow-up mandatory NPI and identify effects of intervention categories. To analyse regional NPI, we accounted for weekday effects by computing relative changes of mean mobility metrics for each weekday compared with the mobility for the same weekday in a reference period (see Supplement, section B for formulae to apply the reference period and normalise the data. This normalisation enabled comparison of mobility on consecutive days, which was necessary for the synthetic difference-in-differences approach (described later) [21].

### Analysis of national interventions

We identified national interventions that comprised non-mandatory measures that were later made mandatory. We used before–after analysis to analyse the effects of the interventions. Although the before–after analysis has limitations, including the lack of controls, it is useful to understand mobility trends when interventions are enacted. We compared the following scores for an intervention on day t:


$$WeekBefore_{t}\;=\frac{1}{7}\;\sum_{i\;=\;t-7}^{t-1}Metric_{i}\;$$

$$WeekAfter_{t}\;=\frac{1}{7}\;\sum_{i\;=\;t}^{t+6}Metric_{i}$$

where *Metric_i_* is one of the three mobility metrics on day *i*. We excluded missing metrics from score calculations. When metrics for one day were missing, we computed the means using the 13 preceding and following days instead of 14. If the time frames for *WeekBefore_t_* or *WeekAfter_t_* coincided with other NPI, the time frame was shortened to avoid overlapping. The weekly mobility change (*WeekMobilityChange_t_*) was calculated as a relative change:


$$WeekMobilityChange_{t}=\frac{WeekAfter_{t}\;-\;WeekBefore_{t}}{WeekBefore_{t}}\;*100$$

As a control analysis, we calculate *WeekMobilityChange_t_* for a date *t* before the intervention, to understand previous regional mobility trends. Since the time frame for *WeekBefore_t_* and *WeekAfter_t_* may differ, we analysed the sensitivity of the results to the window size for some interventions, reported in Supplementary Table S2.

We identified three national interventions in December 2021. The first comprised only non-mandatory measures, the second comprised mostly non-mandatory measures, while the last included mandatory measures. The interventions and time frames for *WeekBefore* and *WeekAfter* are listed below.

1. 26 November 2021: Control time point with no interventions implemented (*WeekBefore*= 19–25 Nov, *WeekAfter=*26 Nov–2 Dec);
2. 3 December 2021: Recommendation to work from home and reduce close contacts (*WeekBefore*= 26 Nov–2 Dec, *WeekAfter* = 3 Dec–8 Dec);
3. 9 December 2021: Recommendation to work from home more, regulations for events, alcohol serving ban after midnight, face mask requirement indoors (*WeekBefore*=3–8 Dec, *WeekAfter*= 9–14 Dec);
4. 15 December 2021: Mandatory work from home, alcohol serving ban, required digital teaching for universities, continued face mask requirement (*WeekBefore*=9–14 Dec, *WeekAfter* = 2–8 Jan).

We analysed the effects of the interventions nationally, in the cities mentioned earlier and in less populated regions. Less populated areas were defined as the half of each county’s municipalities with the least Telenor users on 24 January 2021 (except Oslo, which has one municipality). See Supplement section E for the municipalities and population sizes included in each region. 

### Analysis of regional interventions

We identified regional interventions that started as non-mandatory measures and were later made mandatory, and we analysed mobility changes using synthetic difference-in-differences (SDID) [21]. The SDID analysis compared trends in control locations (called control units) and time periods (called time units) against trends in a region with a new intervention (called treated unit) to assess the NPI’s effect on mobility. We used penalised least squares regression to find a weighted average of control and time units with a pre-treatment trend parallel to the treated unit trend. Then, we used these weights for the control and time units in a two-way fixed effects regression to estimate the average causal effect of adding the intervention. One major assumption of the approach was that no other interventions explain trends in the control regions. No major interventions had been added in the control regions during the weeks around the NPI. The *synthdid* R package was used [21].

We applied SDID for interventions on 28 October and 9 November 2021 with the Tromsø municipality as the treatment region and Bodø, Harstad and Trondheim municipalities as control regions. We chose Bodø, Nordland county’s biggest city, and Harstad, the second most populated municipality in the Troms and Finnmark counties in October 2021 [22]. Trondheim was included to match Tromsø’s urban nature.

We applied SDID for 5 and 8 August 2021 with Bergen as the treatment region and Oslo, Stavanger and Trondheim as controls to match Bergen’s urban nature. We applied SDID for 2 and 24 November 2021 with Trondheim as the treatment region and Oslo, Stavanger and Bergen as controls to match Trondheim’s urban nature.

### Linear regression

We implemented three regression models to study associations between *WeekMobilityChange_t_* and the implementation of multiple intervention categories. We trained the models with data from non-holiday weeks during 2021 for each region at time points when national, local, or no interventions were introduced.

Each model included 52 time points when interventions were added and 37 mid-points of 2-week periods with no new interventions between 24 January and 31 December 2021. Section F in the Supplement includes a mapping of interventions to implementation dates, the algorithm to identify non-intervention time points, and time points with modified comparison time frames (Supplementary Table S10).

We used 19 covariates representing intervention categories, denoted by *X_i,r,t_* which are binary indicators for whether intervention category *i* was implemented on date *t* in region *r, r ∈ R, t ∈ T_r_, i ∈* [1, n] where *n* is the number of intervention categories. The dependent variable was *Y_r,t_*, the log difference between the mobility of the week before and after an intervention on day *t* in region *r.*

Let *𝛽* = (*𝛽_0_*, *𝛽*_1_, …, *𝛽*_n-1_, *𝛽*_n_) be the parameters to estimate and *T_r_* be the intervention and control time points for region *r*. We estimated these coefficients of the following regression equation


$$Y_{r,t}=\beta_{0}+\sum_{i=1}^{n}\beta_{i}*X_{i,r,t}+\varepsilon_{r,t}\;for\;r\in R,\;t\in T_{r}\;$$

More details are appended in Section F of the Supplement. We reported statistically significant coefficients for the covariates most associated with *WeekMobilityChange_t_* for meanDistAway, timeAway and maxDistAway.

## Results

### Visualisation of mobility metrics

**Figure 1:**
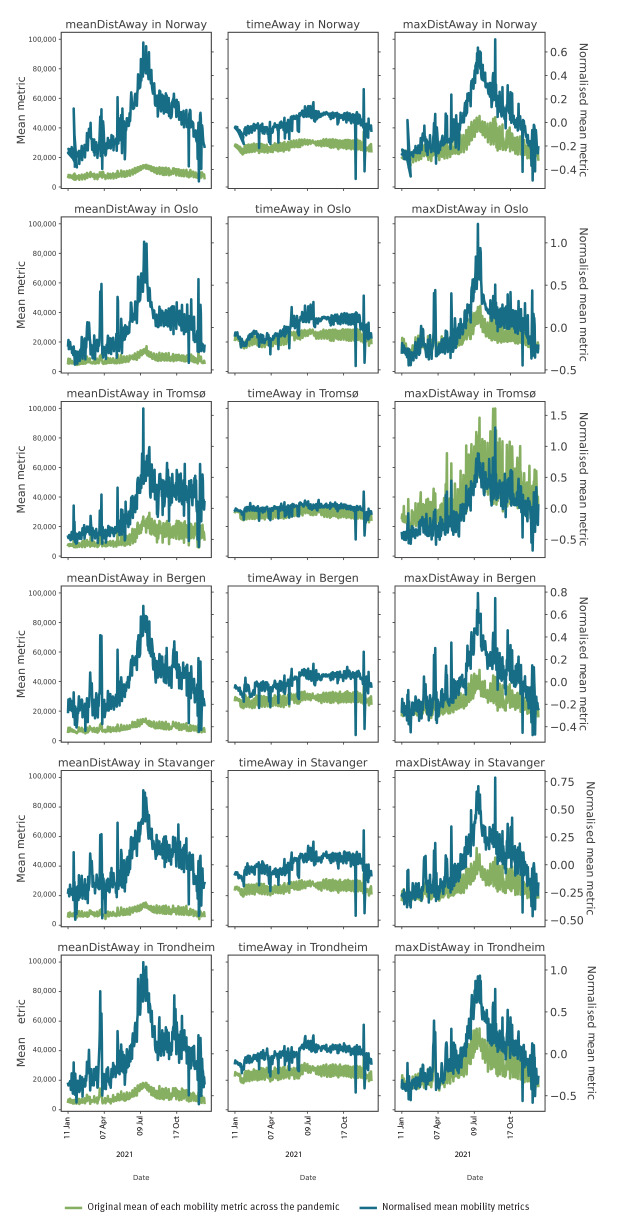
Original and normalised mean mobility metrics, Norway and selected cities, 2021

Figure 1 includes plots of daily mean mobility metrics over 2021 and the normalised and raw mean metrics generally correlate to each other. 

### Overview of the analysis of national and regional interventions

Table 2 provides an overview of our results. Distance and time away from home decreased after initial national non-mandatory measures in December 2021 in Norway. TimeAway further decreased after follow-up mandates. In urban areas, all metrics decreased after follow-up mandates, while in rural areas, only timeAway was reduced. In major cities, none of the metrics changed after non-mandatory regional measures, but time and distance travelled decreased after follow-up regional mandatory measures. In rural regions, only meanDistAway decreased following initial regional measures, and timeAway declined after follow-up regional mandates.

**Table 2:** Summary of effects of national and regional non-mandatory recommendations and follow-up mandates on mobility in more and less densely populated areas, Norway, 2021

Mobility metric	National interventions				Regional interventions			
	Major cities (Bergen, Trondheim, Oslo)		Less populated regions + overall		Major cities (Bergen, Trondheim)		Less populated regions (Tromsø)	
	NM	Follow-up mandate	NM	Follow-up mandate	NM	Follow-up mandate	NM	Follow-up mandate
meanDistAway	↓	↓	↓	—	—	↓	↓	—
timeAway	↓	↓	↓	↓	—	↓	—	↓
maxDistAway	↓	↓	↓	—	—	—	—	↓

NM: not mandatory; ↓ decrease; — no change.

Cities in each region of analysis are listed in parentheses, except in the case of less populated regions for the national intervention analysis which encompasses many smaller towns. All mobility metrics in all areas declined after national non-mandatory measures. Following regional non-mandatory measures, meanDistAway only decreased in less populated areas. TimeAway effectively declined following both national and regional mandatory measures in all regions but mean time away from home only decreased in more populated areas. The effect of non-mandatory measures and follow-up mandatory measures was not consistent between national and regional interventions.

### National interventions

**Figure 2:**
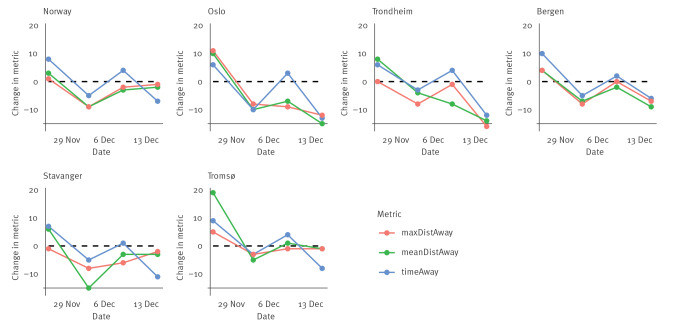
Effects of non-pharmaceutical interventions on mobility nationally and in urban areas, Norway, 2021

Nationally, mobility declined following initial non-mandatory measures (Figure 2). All further measures, including mandates on 15 December, were associated with comparatively small effects on meanDistAway and maxDistAway. However, timeAway further decreased after the 15 December mandates, nationally and in all regions.

The percentage of mobility change in each of the three mobility metrics from the week before to that after different intervention dates is shown for selected regions. The intervention dates include a control time point in November and three interventions in December, with only non-mandatory measures on 3 December, mostly non-mandatory measures on 9 December and follow-up mandatory measures on 15 December. The regions analysed include all of Norway and major cities in Norway.

In Bergen and Trondheim, meanDistAway declined after follow-up mandates more than after initial recommendations. In Tromsø, a less populated city, meanDistAway did not decline after follow-up mandates, while timeAway declined. In Tromsø and Trondheim, meanDistAway declined more after recommendations on 9 December than after initial recommendations on 3 December, while similar reductions in meanDistAway followed both measures in Bergen. It is difficult to interpret results from Stavanger and Oslo, as face masks were made mandatory in both cities and home office became a requirement in Oslo on 3 December.

**Figure 3:**
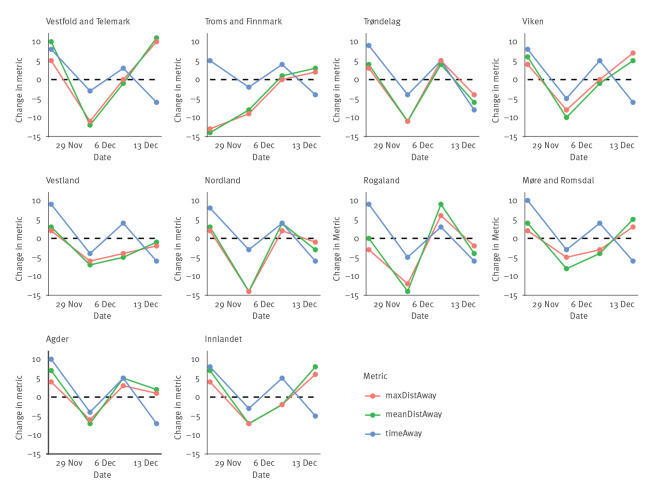
Effects of national non-pharmaceutical interventions on mobility in less populated regions, Norway, 2021

In rural areas, meanDistAway declined after non-mandatory measures, but did not decline further after follow-up mandates (Figure 3). This occurred in all counties except Trøndelag. MeanDistAway was reduced in Nordland, Rogaland and Vestland, but the magnitudes were small compared with the reductions after initial measures.

The percentage of mobility change in each of the three mobility metrics from the week before to the week after different intervention dates is shown for selected regions. The intervention dates include a control time point in November and three interventions in December, with only non-mandatory measures on 3 December, mostly non-mandatory measures on 9 December and mandatory follow-up measures on 15 December.

TimeAway decreased more after mandates than following initial non-mandatory measures in rural areas. These results align with national mobility trends. In all counties, except Vestland and Møre og Romsdal, initial non-mandatory measures on 3 December were followed by the largest mobility reductions, and metrics did not further reduce after implementing the non-mandatory interventions on 9 December.

### Regional interventions

When analysing a pair of non-mandatory and follow-up mandatory measures in Tromsø, both decreased meanDistAway but the former had a larger effect (Table 3). However, only the mandate reduced timeAway and maxDistAway. Non-mandatory measures did not reduce mobility in Bergen and Trondheim as much as further mandates did (Table 3). Weights and plots of mobility trends detected by SDID are provided in the Supplement, section E, and the synthetic trends generally matched trends in regions where interventions were introduced.

**Table 3:** Effects of regional non-pharmaceutical interventions on mobility, Norway, 2021

City	Date	Intervention	meanDistAway	timeAway	maxDistAway
Tromsø	28 Oct	Recommendation to work at home, reduce social contacts, and use face masks	−0.06 (−0.12 to 0.00)	0 (−0.03 to 0.03)	0.01 (−0.15 to 0.17)
	9 Nov	Requirement to work at home and use face masks, many events cancelled	−0.03 (−0.03 to −0.03)	−0.03 (−0.04 to −0.03)	−0.05 (−0.08 to −0.01)
Bergen	5 Aug	Recommendation to use face masks and limit people in homes to 10	−0.01 (−0.21 to 0.18)	0.01 (−0.02 to 0.05)	0.04 (0.03 to 0.05)
	12 Aug	Requirement to use face masks and limit people in homes to 10	−0.04 (−0.05 to −0.03)	−0.02 (−0.06 to 0.02)	−0.05 (−0.10 to −0.01)
Trondheim	2 Nov	Recommendation to use face masks	0.03 (−0.01 to 0.07)	0.01 (−0.00 to 0.02)	−0.02 (−0.05 to 0.01)
	24 Nov	Requirement to use face masks	−0.05 (−0.06 to −0.05)	−0.03 (−0.05 to 0.00)	0.06 (−0.00 to 0.13)

The causal effects of different regional interventions approximated by synthetic difference-in-differences on each of the three mobility metrics is shown, with 95% confidence intervals in parentheses. The interventions analysed comprise pairs of non-mandatory and follow-up mandatory measures from three cities: Tromsø, Bergen and Trondheim. In Bergen and Trondheim, mandatory measures continued to reduce mean distance and time away from home. In Tromsø, a smaller city, initial non-mandatory measures reduced the mean distance away from home. Further mandatory measures mainly affected time away from home.

### Effects of intervention categories

Table 4 shows statistically significant features associated with *WeekMobilityChange* in meanDistAway, timeAway, and maxDistAway. 

**Table 4:** Non-pharmaceutical interventions intervention categories with the largest effects on mobility, Norway, 2021

Intervention category	meanDistAway		timeAway		maxDistAway	
	Coefficient (95% CI)	p value	Coefficient (95% CI)	p value	Coefficient (95% CI)	p value
1 m or 2 m rule implemented	−0.17 (−0.34 to −0.00)	0.047	NS		NS	
Removing recommendation of physical distancing or easing metre rule	NS		−0.06 (−0.11 to 0.01)	0.030	NS	
Gyms reopened	NS		NS		−0.18 (−0.35 to −0.01)	0.037
Restaurants, shops and businesses reopened	NS		0.07 (0.03 to 0.11)	0.001	NS	

NS: not significant at 5% level.

The effect of different intervention categories on the changes in meanDistAway, timeAway and maxDistAway from the week before to that after an intervention are shown. Intervention categories that were not associated with significant effects on any mobility metrics include measures related to teleworking, schools, alcohol serving and face mask requirements. The R-squared values for the models predicting changes in meanDistAway, timeAway and maxDistAway were 0.279, 0.532 and 0.295, respectively.

Stricter metre rules were associated with reductions in meanDistAway. Restaurant and shop re-openings were associated with timeAway increasing, while easing physical distancing measures was associated with reductions in timeAway. In addition, reopening gyms was correlated with reductions in maxDistAway but not in meanDistAway. Interventions related to teleworking, schools, alcohol serving and face mask requirements were not associated with any significant mobility changes.

## Discussion

We found that distance and time away from home decreased after initial national non-mandatory measures in December 2021 in Norway. Time travelled further decreased after follow-up mandates. Interestingly, after follow-up mandates, all metrics decreased in urban areas, while only time travelled decreased in rural areas. Analysis of separate regional interventions provided overall similar results as for national measures, with some differences. For regional interventions, we observed that distance decreased more in large cities after follow-up mandates than after initial recommendations. Mobility did not reduce following regional non-mandatory measures in urban areas but did decrease after follow-up mandates. In summary, distance and time travelled decreased following non-mandatory national measures, while after regional non-mandatory measures, distance only decreased in rural areas. Following mandatory follow-up measures, time travelled declined further, especially in urban areas, where distance travelled was further minimised.

Moreover, we observed higher relative distance and time travelled reductions in urban areas. This result aligns with findings on mobility reduction from mandatory physical distancing policies in the UK and in 10 Western Pacific countries and areas, which identified greater mobility reductions in areas with high population density [23,24]. One explanation is that living in rural regions is associated with considerable everyday mobility. Workplaces, retail facilities and key services are often located far from home, and a higher percentage of Norwegians in rural areas compared with those in urban areas rely on driving to reach destinations [25]. In contrast, city residents live closer to workplaces and shops and have better pick-up and delivery options, which could explain the more negligible effect on distance metrics for rural vs urban inhabitants.

Time travelled decreased more after follow-up mandates than after non-mandatory measures in all cases. One plausible explanation is mandatory teleworking, which increases time spent at home for those noncompliant with initial recommendations. Since distance travelled did not decline after follow-up mandates in less populated areas, non-mandatory measures, which are less costly and invasive, may be more appropriate to generate compliance. Strong public trust in the government may explain the effectiveness of non-mandatory measures in Norway [26,27].

In Tromsø and Trondheim, the second set of recommendations in December 2021 was more impactful than initial measures in March 2021, while the recommendations had similar effects in Bergen. This finding could be because Tromsø and Trondheim, in contrast with Bergen, had recent peaks in COVID-19 cases. Aware of declining numbers of cases, the population may have had a lower perceived risk, so non-mandatory measures may have been less effective [28]. Moreover, Tromsø recommended face masks before 3 December 2021, which can explain why the initial 3 December interventions only minimally affected mobility compared with the later interventions. This varying effect of recommendations indicates that recent COVID-19 case trends should be analysed before introducing such measures.

We also identified the effect of isolated intervention categories on mobility by multivariate regression analysis. Stricter metre rules were associated with decreased distance travelled. Reduced physical distancing measures were correlated with decreased time travelled, perhaps because people feel safer with recommendations in place. Gyms reopening was associated with decreased maxDistAway, possibly because people were training outside and further away from their homes when gyms were closed. Further investigation is necessary to determine the validity of interventions that did not have significant effects in our analysis. NPI with non-significant effects on all metrics included measures related to teleworking, schools, alcohol serving and face masks.

To our knowledge, our study is the first of this size to analyse the effects of COVID-19 interventions on both distance and time away from home, which should be analysed together as they offer different insights into human behaviour. We found that interventions affected distance and time travelled differently. Nationally, follow-up mandates impacted time more than distance, and these metrics were influenced by different interventions: stricter metre rules and reopening of gyms influenced distance, while reopening of restaurants and shops and easing physical distancing affected time. Future work should investigate which metric is more relevant to COVID-19 transmission and if interventions should be designed to reduce one metric.

This observational study has many limitations. With the before–after analysis, potential confounders include temperature and weather. While we analysed mobility trends before interventions were enacted, there were no controls. Yet, because we studied short periods before and after NPI, it is reasonable to assume that the differences in the absence of interventions were constant. Results from the before–after analysis should be interpreted as associations and not as cause and effect. For the SDID approach, a case–control analysis, it was difficult to choose appropriate control regions. However, our hypothesis about the varying regional effect of follow-up mandates was supported by analyses using different interventions and two methodological approaches, increasing confidence in our conclusions.

The interventions identified in our study should be further validated by comparison with public intervention trackers, collected for instance through news sources or public health officials. We did not assess how interventions impacted COVID-19 incidence, owing to factors such as under-reporting of cases and delays between transmission and testing positive for COVID-19 [29]. When connecting our results to understanding effects on transmission, we note that mobility and transmission were not always correlated, especially during later waves of the pandemic [30]. Mobility serves as an early signal for the effects of NPI. However, limitations include that mobility is only a proxy for a reduction in contact rate and there may be a selection bias in mobile phone use and ownership. However, two studies found that this bias does not drastically affect mobility estimates [31,32]. We also lack mobility data for 1 day approximately every 3 weeks, due to data anonymisation. In addition, some interventions were implemented shortly after others, with only 6 days between the two interventions. As a result of data anonymisation and shorter intervals between some interventions, some comparison frames before and after an intervention had a different number of days or different days of the week. Hence, the metrics could be affected by day-of-the-week effects in these cases.

Our results are for Norway but may be relevant for other countries. Norway’s population has high trust in the government and local authorities [26,27]. This could be similar in other countries, especially in the Nordic countries. However, future studies are necessary to assess whether our results are transferable to other populations and settings.

## Conclusions

Nationally, mobility declined following initial non-mandatory measures in all cities and counties studied. Distance travelled decreased following non-mandatory measures in less populated areas and declined further after follow-up mandates in more populated areas. We found that stricter metre rules, reopening of gyms and of restaurants and shops were significantly associated with changes in mobility. These observations have important policy implications on which NPI to implement, the choice between non-mandatory or mandatory measures, and using regional or national interventions. Since distance travelled declined less after follow-up mandates than after initial recommendations in less populated areas, less invasive and costly non-mandatory measures may be sufficiently effective for rural areas in the case of Norway.

## Supplementary Data

1409000 Supplement
