# Supplementary material for: Effects of non-compulsory and mandatory COVID-19 interventions on travel distance and time away from home, Norway, 2021
Source: Euro Surveill. 2023 Apr 27;28(17):2200382. doi: 10.2807/1560-7917.ES.2023.28.17.2200382 (PMC10283475; doi:10.2807/1560-7917.ES.2023.28.17.2200382)
Supplement: Supplement [file 22-00382_KAMINENI_Supplement.pdf]

Supplementary material: Effects of non-compulsory and mandatory COVID-19 interventions on travel distance and time away from home, Norway, 2021

This supplementary material is hosted by Eurosurveillance as supporting information alongside the article "Effects of non-compulsory and mandatory COVID-19 interventions on travel distance and time away from home, Norway, 2021", on behalf of the authors, who remain responsible for the accuracy and appropriateness of the content. The same standards for ethics, copyright, attributions and permissions as for the article apply. Supplements are not edited by Eurosurveillance and the journal is not responsible for the maintenance of any links or email addresses provided therein.

A. Aggregated Mobility Data

The analysis in this work is based on mobile phone data of all users in Telenor Norway's network. The exact composition of the user population vs. the total Norwegian population is not known to the authors of this paper, and this contributes a potential source of bias. However, Telenor's sizable market share (47.5% per 2019) indicates that the data are quite representative. The data is acquired in the following way: Each phone that is connected to the mobile network will, at any given time, be connected to one single base station. Each base station has a known coverage area, and this coverage area is used to determine a fixed coordinate position that is used as a proxy for the position of all phones connected to said base station. Thus, each base station gets mapped to one single geographical point (there is no triangulation of phone position between base stations involved), and this defines the geospatial resolution of our dataset. When a phone is moving in space, its connection may be handed over from one base station to another, and this handover event is timestamped and logged. Thus, the coordinate position of each phone is updated every time it moves to a new grid point. The log may also be updated by phones not changing base stations, if they have been idly connected for a period. The list of all time stamped events for each connected phone serves as the basis for the data set used in this paper. The coverage area of a cell tower varies significantly, from the order of tens of metres in dense urban areas to kilometres in remote, uninhabited areas.

For the analyses presented here, the data are aggregated into three distinct measures: radius of gyration, time away from home, and maximum distance away from home. Each of these metrics is calculated for each individual subscriber each day and is then aggregated into an empirical distribution for each municipality and each day, represented as quantiles. This aggregation is performed automatically inside the mobile network operator's facilities, immediately after data acquisition. The resulting distributions are the only information that is stored. Data is not available approximately one day every three weeks due to anonymization purposes. On these days, all pseudonyms that identify individuals are replaced, and since individuals' homes and movements thus cannot be linked, data is not reported. A day is defined as 04:00 to 04:00 the next day, as we expect most people to be asleep/at home at 04:00. There could be some small effect of people being misassigned from going out, but we believe the effect to be small.

I. Radius of Gyration

The radius of gyration for one individual for a day is defined by the formula

$$R_g = \sqrt{\frac{\sum_{i=1}^{N} m_i(\vec{r}_i - \vec{r_C})^2}{\sum_{i=I}^{N} m_i}}$$

Here, $\vec{r}_i$ contains the coordinates (using the coordinate system UTM33) of the ith visited place of an individual, while $\vec{r}_C$is the "centre of mass", $\vec{r}_C = \sum_i m_i\vec{r}_i \,/\, \sum_i m_i$. For meanDistAway, the temporal duration of stay at each $\vec{r}_i$was used as the weighting parameter m_i. Individuals are assigned to a municipality based on where they were connected at 04:00., i.e. where they presumably spent the night. N is the number of locations visited and is different for every individual and day.

II. Time Away From home

The time away from home is defined as the amount of time a subscriber spends connected to a cell tower other than their home tower. Their home tower is taken to be where they were connected at 04:00., i.e. where they presumably spent the night.

III. Max Distance Away From home

The max distance away from home is taken as the maximum Euclidean distance between the home tower (as defined in the previous paragraph) and all other towers to which the subscriber is connected during the day.

B. Normalisation of Mobility Data

We compute the mean of each metric for each day of the week during a three-week reference period from September 2 to 23, 2021 when mobility levels were stable, and few interventions were in place. Let dayOfWeek be Monday, Tuesday, … or Sunday. Then we have

$$RefMeanDistAway_{dayOfWeek} = \frac{\sum_{t\,=\,Sept\,2}^{Sept\,23} meanDistAway_t * 1_{t\ is\ dayOfWeek}}{\sum_{t\,=\,Sept\,2}^{Sept\,23} 1_{t\ is\ dayOfWeek}}$$

$$RefTimeAway_{dayOfWeek} = \frac{\sum_{t\,=\,Sept\,2}^{Sept\,23} timeAway_t * 1_{t\ is\ dayOfWeek}}{\sum_{t\,=\,Sept\,2}^{Sept\,23} 1_{t\ is\ dayOfWeek}}$$

$$RefMaxDistAway_{dayOfWeek} = \frac{\sum_{t\,=\,Sept\,2}^{Sept\,23} maxDistAway_t * 1_{t\ is\ dayOfWeek}}{\sum_{t\,=\,Sept\,2}^{Sept\,23} 1_{t\ is\ dayOfWeek}}$$

Then, on each day t and for each metric, we computed the relative change of the mean for each day of the week compared to the mean of the same mobility metric during the same weekday in the reference period. Let dayOfWeek(t) be the day of the week of day t.

$$NormMeanDistAway_t = \frac{meanDistAway_t - RefMeanDistAway_{dayOfWeek(t)}}{RefMeanDistAway_{dayOfWeek(t)}}$$

$$NormTimeAway_t = \frac{timeAway_t - RefTimeAway_{dayOfWeek(t)}}{RefTimeAway_{dayOfWeek(t)}}$$

$$NormMaxDistAway_t = \frac{maxDistAway_t - RefMaxDistAway_{dayOfWeek(t)}}{RefMaxDistAway_{dayOfWeek(t)}}$$

We utilised these relative mobility values in the synthetic difference-in-differences (SDID) approach, as metrics for consecutive days must be comparable with each other. This normalisation is not necessary for the before-after analysis, as the time periods of comparison are weekly units, rather than daily units as in the SDID analysis, so weekday variation will not affect the analysis.

C. Map of Norway

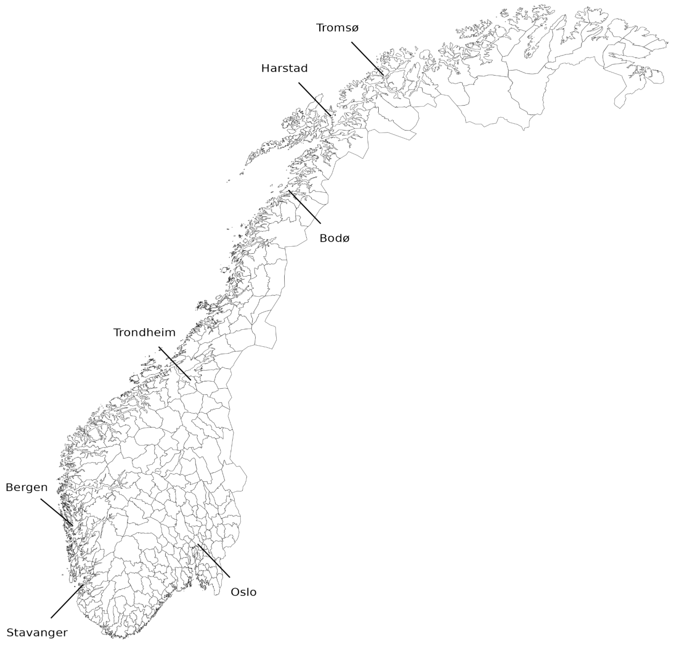

Figure S1. Map of Norway with selected cities labelled
Cities used in analyses of national and local interventions are labelled. Cities included as control regions for the SDID analyses are also included.

D. National and Local Interventions

Table S1. Non-pharmaceutical interventions in Norway, Oslo, Bergen, Trondheim, Tromsø, and Stavanger from February 1, 2021 to December 31, 2021 and Coverage of Mobility Data Available in the Weeks Before and After Interventions
This list does not include entry restrictions into the country from abroad or vaccine interventions.
** = These interventions are not included in any analyses due to limited mobility data before or after the intervention, but they are listed here for completeness purposes. l

Geographical Area	Date of Effect	Summary of Action	# of Days with Mobility Data in Week Before	# of Days with Mobility Data in Week After
Norway	February 3	More people are allowed in private homes, and event restrictions are eased. [1]	7	7
	March 25	A 2-metre rule and face masks are recommended. Alcohol is prohibited, and more restrictions are placed on events. [2]	7	7
	April 16	Alcohol, event, school, and private home restrictions are eased in the 1st phase of reopening. [3]	7	6
	May 27	Alcohol and event restrictions are eased in the 2nd phase of reopening. [4]	7	7

	June 20	Alcohol, event, work-from-home, and private home restrictions are eased in the third phase of reopening, along with the gradual reopening of the travel network. [5]	7	6
	September 4	Event restrictions are eased. [6]	7	3
	September 25	Alcohol and event restrictions are eased, social distancing recommendations are removed. [7]	7	5
	December 3	Work from home and reducing close contacts is recommended. [8]	7	5
	December 9	Recommendations to work from home even more are added, along with initial alcohol, event, and face mask restrictions. [9]	5	6
	December 15	Work from home becomes mandatory, and alcohol is prohibited. Universities are required to do digital teaching, and the requirement to wear face masks indoors is extended to indoor events, libraries, museums, etc. [10]	6	7
Oslo	February 3	Restaurants and shops can reopen, and kindergartens, primary and secondary schools will change from red to yellow risk levels. [11]	7	7
	March 2**	Restaurants and shops are closed, and event, private home, and school restrictions are added. [12]	7	4
	April 19	School restrictions are eased. [13]	7	6
	May 6	Restaurants and shops can reopen.[14]	7	6
	May 26	Restrictions on alcohol and events are eased, and restaurants and shops can reopen further. [15]	7	7
	June 16	2-metre rule and alcohol, event, and private home limits are eased. [16]	7	6
	July 5	Working from home and alcohol restrictions are eased.[17]	7	7
	November 24**	Face masks are recommended. [18]	4	7
	December 15	Red and yellow levels are added for kindergartens and schools in Oslo. [19]	6	7
Bergen	February 7	Alcohol, event, school, work from home, and private home restrictions are added, and face masks are required. [20]	7	7
	February 21	Alcohol, event, school, work from home, and private home restrictions are eased. [21]	6	7

	March 27 **	Gyms are closed. [22]	7	3
	March 31**	Gyms opened. [23]	3	7
	April 19	Event, school, and private home restrictions are added, and face masks are required. [24]	7	6
	May 12	Alcohol restrictions are tightened, but private home restrictions are eased. [25]	6	7
	May 31	Event, alcohol, work from home, and private home restrictions are eased. [26]	7	7
	August 5	Private home limits up until 10 people and face masks are recommended. [27]	6	7
	August 12	Private home limits up until 10 people and face masks are made mandatory. [28]	7	7
	September 1	Private home and face mask restrictions are eased. [29]	7	5
	December 9	Face masks are required. [30]	5	6
	December 15	Schools become digital. [31]	6	7
Trondheim	February 2	Alcohol restrictions are eased. [32]	7	7
	February 10	Alcohol and work-from-home restrictions are added, and some businesses are closed. [33]	7	6
	May 19	Face masks are required. [34]	7	7
	June 1	Alcohol, private home, and work from home restrictions are added. [35]	7	7
	June 22	Alcohol, private home, work from home, and face mask restrictions are eased. [36]	6	7
	August 26	Face masks are required.[37]	7	7
	September 2**	Events are more restricted, as NTNU recommends smaller student events. [38]	7	4
	September 21	Face mask requirement is eased. [39]	7	7
	November 2	Face masks are recommended. [40]	7	6

	November 24**	Face masks are required. [41]	4	7
Tromsø	March 5**	Face masks are required, gyms are closed, and private home restrictions are added. [42]	7	4
	March 19	Face mask and private home restrictions are eased, and gyms re-opened. [42]	7	7
	July 2	Face masks are recommended. [43]	7	7
	July 13	Face mask recommendation is eased.[44]	6	7
	Oct 28	Recommendations to work from home more, reduce social contacts, use face masks, and follow the 1-metre rule are added. [45]	6	6
	November 9	Mandatory work from home, obligations to use face masks, and event restrictions are added. [46]	6	6
	November 30	Some face mask and event restrictions are eased. [47]	7	6
Stavanger	April 16	Event restrictions and mandatory home office are added. [48]	7	6
	May 6	Work from home, alcohol, and event restrictions are eased. [49]	7	6
	July 8	Event restrictions are eased. [50]	7	6
	September 1	Face masks are required in public transport and indoors when not possible to keep a metre distance. [51]	7	5
	September 15**	Face mask restrictions are eased. [51]	4	7
	December 2	Face masks are required. [52]	7	6
	December 9	School restrictions are added. [53]	5	6
	December 15	School restrictions are added. [54]	6	7

I. Checking Effects of Shorter Window Sizes

We checked the effects of shorter window sizes for a few interventions, specifically the December 3, 9, and 15 national interventions. In Table S2, we compared the results we reported with unequal window sizes with the results we obtain when we use the same length window size for the week before and the week after each intervention. The same length window size is obtained by taking the shortest window length of the week before and after each

intervention. From observation, the results with the same window lengths do not differ much from the original results, and the interpretation would be the same. However, the lack of consistent window length through the results is still an important limitation.

Table S2. Effects of Shorter Window Sizes on Results for National Interventions. The original results based on varying window sizes for weeks before and after an intervention that we report in the main manuscript are included as the main values in the table. The results we obtain from using the same length window sizes are in parentheses. Differences of more than two percentage points are highlighted in light red.

Metric	Region	Intervention Date (t)		
		3 December	9 December	15 December
meanDistAway	Norway	-9 (-9)	-3 (-2)	-2 (2)
	Oslo	-10 (-7)	-7 (-6)	-15 (-14)
	Trondheim	-4 (-5)	-8 (-7)	-14 (-10)
	Bergen	-7 (-7)	-2 (-2)	-9 (-7)
	Stavanger	-15 (-15)	-3 (-1)	-3 (0)
	Tromsø	-5 (-6)	1 (5)	-1 (4)
timeAway	Norway	-5 (-5)	4 (4)	-7 (-6)
	Oslo	-10 (-10)	3 (4)	-13 (-12)
	Trondheim	-3 (-5)	4 (4)	-12 (-10)
	Bergen	-5 (-5)	2 (2)	-6 (-4)
	Stavanger	-5 (-5)	1 (2)	-11 (-10)
	Tromsø	-3 (-3)	4 (4)	-8 (-7)
maxDistAway	Norway	-9 (-7)	-2 (-1)	-1 (2)

	Oslo	-8 (-5)	-9 (-6)	-12 (-12)
	Trondheim	-8 (-7)	-1 (0)	-16 (-13)
	Bergen	-8 (-6)	0 (2)	-7 (-4)
	Stavanger	-8 (-8)	-6 (-4)	-2 (0)
	Tromsø	-3 (-2)	-1 (3)	-1 (3)

E. Non-compulsory Measures and Mandates

I. SDID Weights and Plots of Trends for Regional Interventions

In order to compare trends between the control regions and the region with the intervention (referred to as the treated unit), we used SDID to calculate weights for each control region to create a weighted average of the control regions that is as similar as possible to the treated unit. We present the weights for each control region for each application of SDID to improve interpretability. In addition, we created plots comparing the trend in the synthetic control, or the weighted average of the controls, and in the treated unit.

Table S3. SDID Weights for Analysing Tromsø Interventions. The weights assigned to each control region are presented for the applications of SDID to estimate the effect of the two interventions in Tromsø on each of the three mobility metrics.

Date	Control Region	meanDistAway	timeAway	maxDistAway
Oct 28	Bodø	0.329	0.349	0.303
	Harstad	0.343	0.320	0.318
	Trondheim	0.328	0.331	0.379
Nov 9	Bodø	0.332	0.277	0.348
	Harstad	0.385	0.364	0.309
	Trondheim	0.282	0.359	0.343

Table S4. Visualisation of Mobility Trends in Synthetic Control versus Treated Regions for Analysing Tromsø Interventions. The normalised mobility metrics are plotted for a week before and after each intervention for both the synthetic control and treated regions. The synthetic control is a weighted average of the control regions, where the weights are chosen to minimise the difference between the synthetic control and treated region's mobility trends before the intervention.

	10/28 - Non-Compulsory Measures	11/9 - Mandate
Mean Dist Away	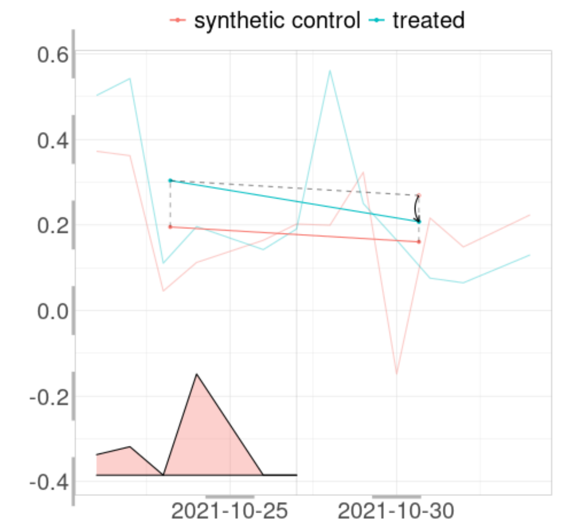	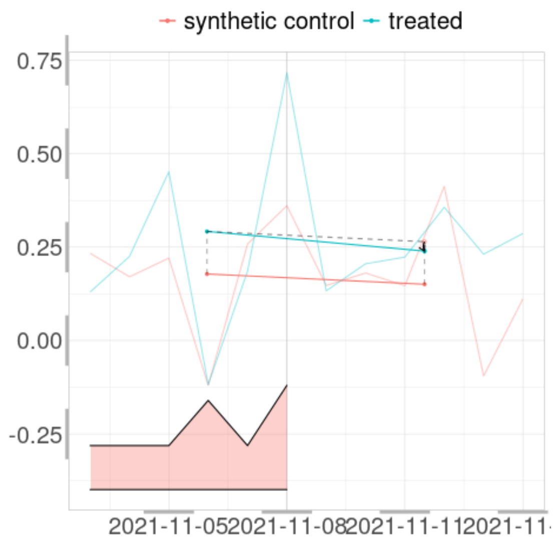
Time Away	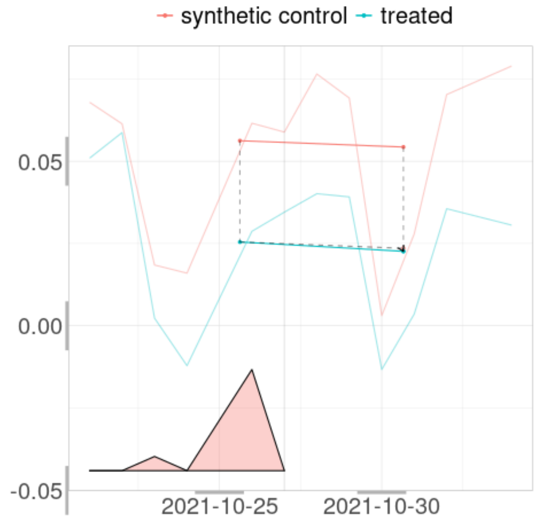	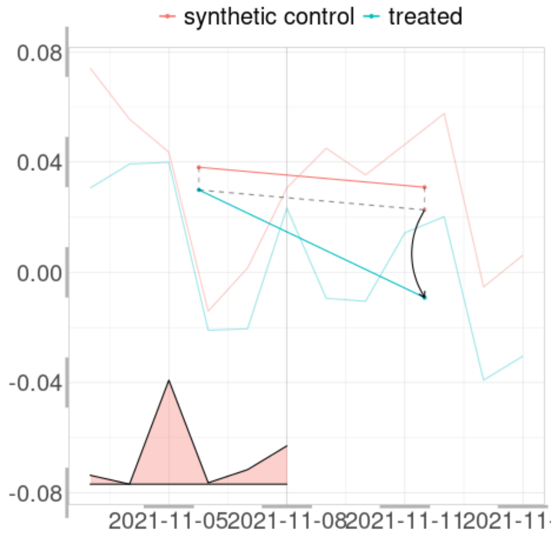

Max Dist Away	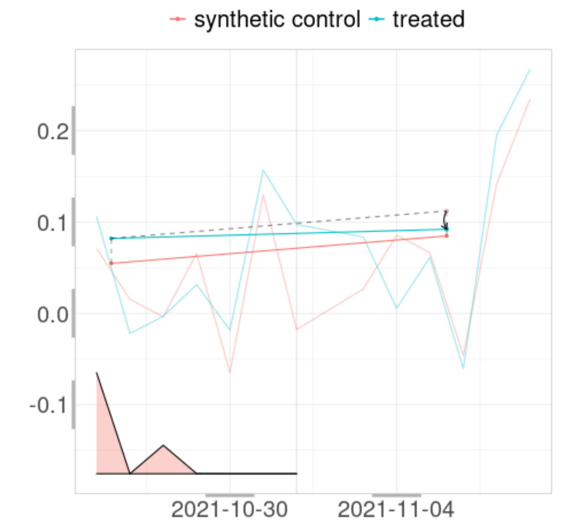	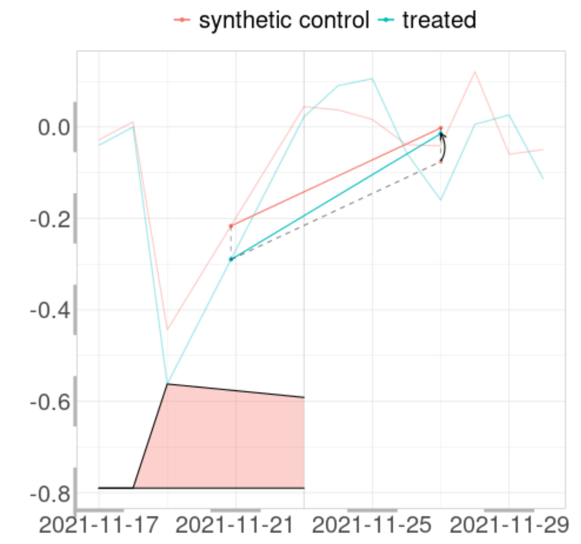

Table S5. SDID Weights for Analysing Bergen Interventions. The weights assigned to each control region are presented for the applications of SDID to estimate the effect of the two interventions in Bergen on each of the three mobility metrics.

Date	Control Region	meanDistAway	timeAway	maxDistAway
Aug 5	Oslo	0.405	0.334	0.376
	Trondheim	0.327	0.324	0.332
	Stavanger	0.268	0.342	0.293
Aug 12	Oslo	0.397	0.321	0.376
	Trondheim	0.303	0.349	0.288
	Stavanger	0.300	0.330	0.336

Table S6. Visualisation of Mobility Trends in Synthetic Control versus Treated Regions for Analysing Bergen Interventions. The normalised mobility metrics are plotted for a week before and after each intervention for both the synthetic control and treated regions. The synthetic control is a weighted average of the control regions, where the weights are chosen to minimise the difference between the synthetic control and treated region's mobility trends before the intervention.

	8/5 - Non-Compulsory Measures	8/12 - Mandate

<table>
<tr><td>Mean Dist Away</td><td>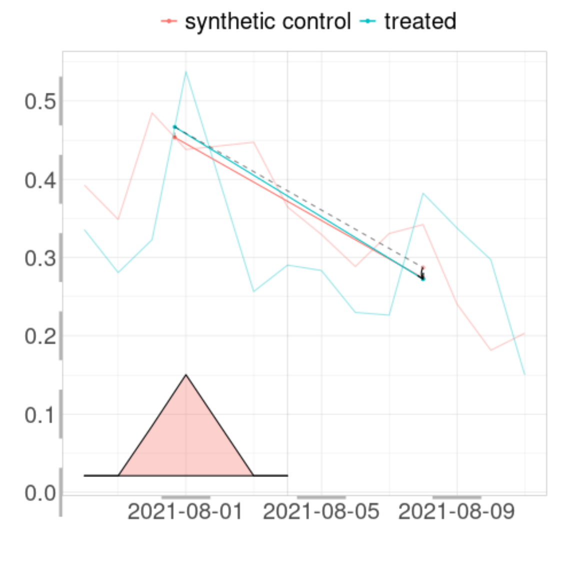
</td><td>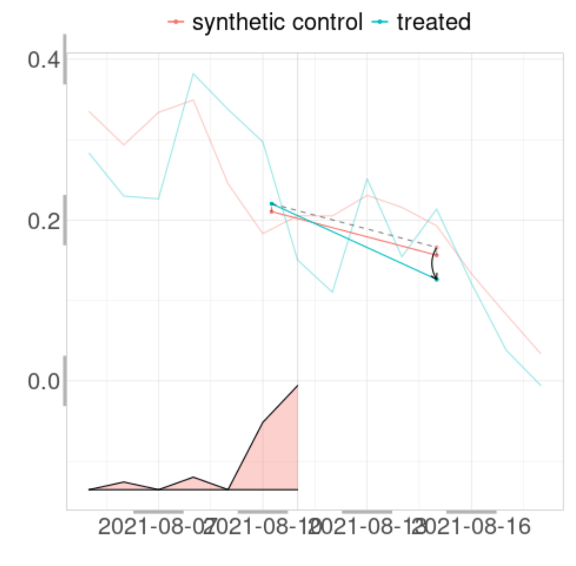
</td></tr>
<tr><td>Time Away</td><td>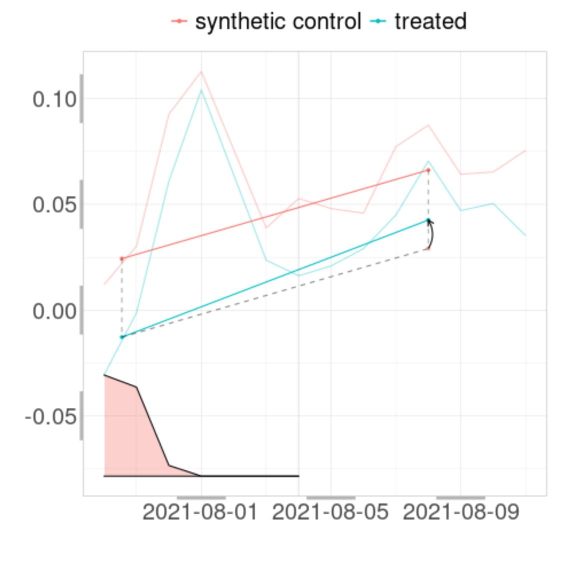
</td><td>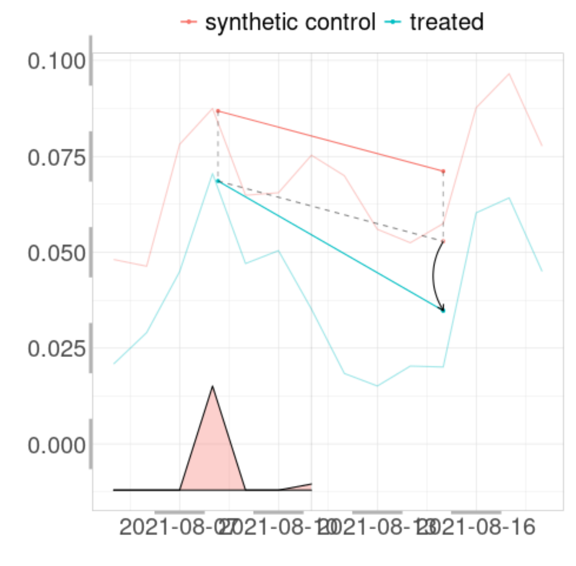
</td></tr>
</table>

Max Dist Away	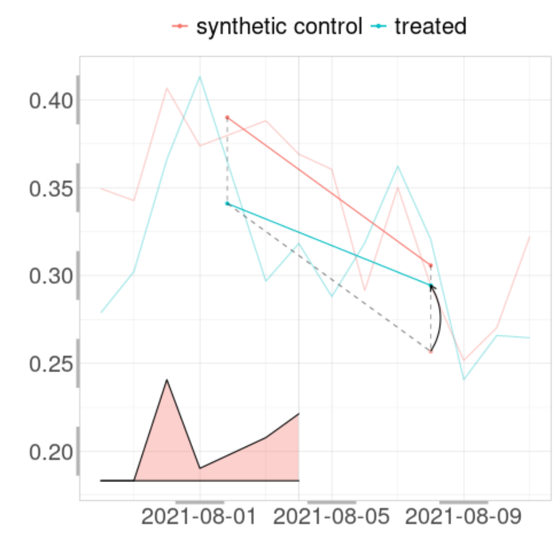	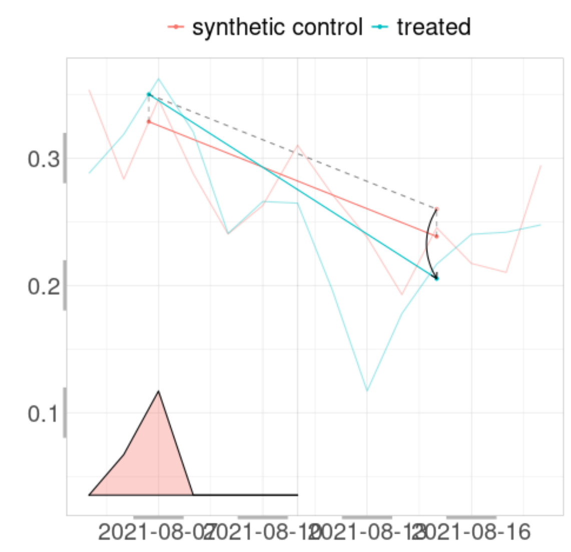

Table S7. SDID Weights for Analysing Trondheim Interventions. The weights assigned to each control region are presented for the applications of SDID to estimate the effect of the two interventions in Trondheim on each of the three mobility metrics.

Date	Control Region	meanDistAway	timeAway	maxDistAway
Nov 2	Oslo	0.494	0.301	0.347
	Bergen	0.337	0.344	0.357
	Stavanger	0.169	0.355	0.296
Nov 24	Oslo	0.336	0.332	0.334
	Bergen	0.330	0.334	0.332
	Stavanger	0.334	0.334	0.334

Table S8. Visualisation of Mobility Trends in Synthetic Control versus Treated Regions for Analysing Trondheim Interventions. The normalised mobility metrics are plotted for a week before and after each intervention for both the synthetic control and treated regions. The synthetic control is a weighted average of the control regions, where the weights are chosen to minimise the difference between the synthetic control and treated region's mobility trends before the intervention.

	11/2 - Non-Compulsory Measures	11/24 - Mandate

Mean Dist Away	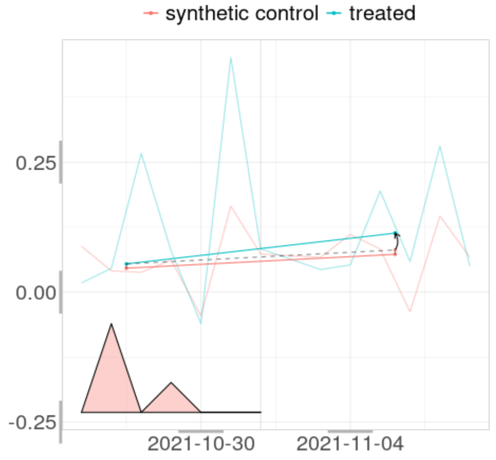	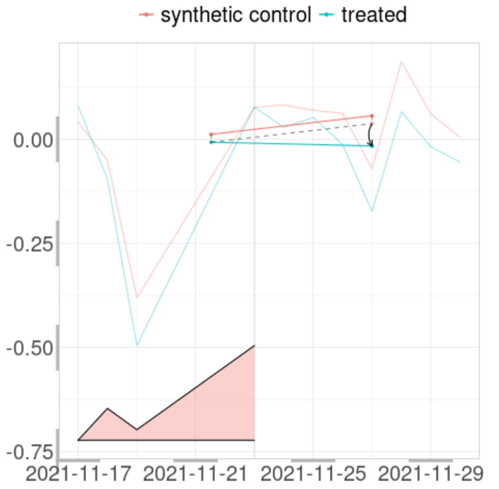
Time Away	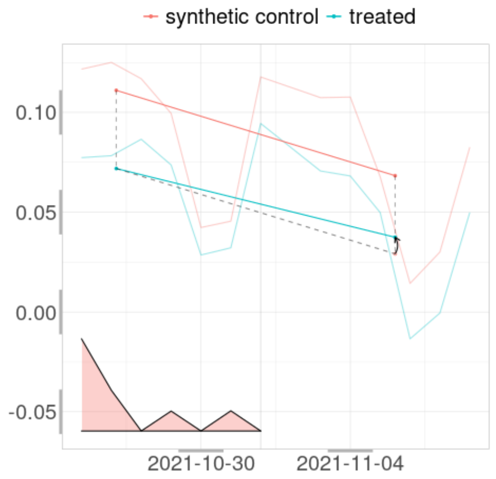	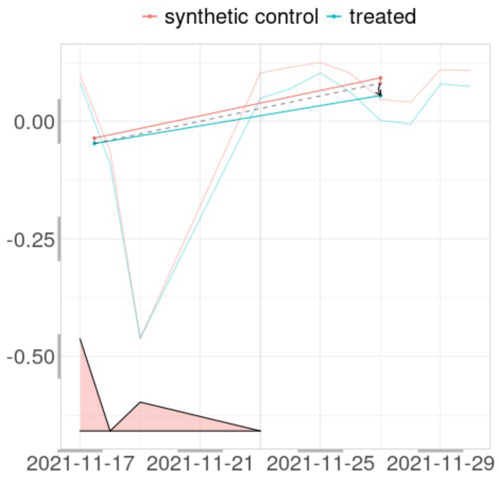
Max Dist Away	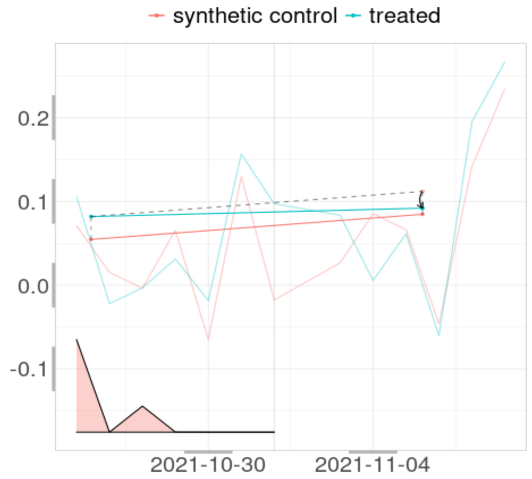	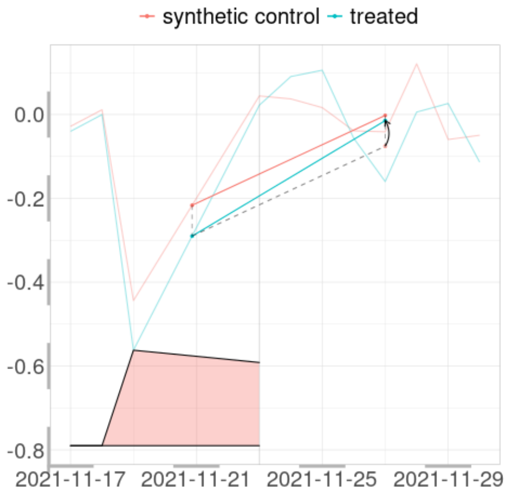

II. Least Populated Municipalities in Each County

The least populated municipalities were chosen based on the number of Telenor users on January 24, 2021. The number of users vary slightly between days.

Table S9. List of Municipalities with Smallest Number of Telenor Users in each Fylke in Norway.

Fylke	Total Number of Telenor Users in Regions Considered	Municipalities
Rogaland	36048	Utsira, Kvitsøy, Bokn, Hjelmeland, Sokndal, Bjerkreim, Lund, Suldal, Sauda, Randaberg, Strand
Møre og Romsdal	57709	Smøla, Sande, Tingvoll, Aukra, Gjemnes, Fjord, Vanylven, Stranda, Hareid, Aure, Averøy, Sykkylven, Surnadal
Nordland	34974	Træna, Vevelstad, Røst, Værøy, Moskenes, Vega, Sømna, Flakstad, Bindal, Rødøy, Herøy, Beiarn, Nesna, Hattfjelldal, Grane, Leirfjord, Evenes, Sørfold, Lurøy, Lødingen
Viken	152600	Aremark, Gjerdrum, Flå, Skiptvet, Marker, Hurdal, Rollag, Hole, Våler, Jevnaker, Hvaler, Nore og Uvdal, Krødsherad, Flesberg, Ål, Enebakk, Råde, Nesbyen, Gol, Rakkestad, Rælingen, Lunner, Hemsedal, Sigdal, Aurskog-Høland
Innlandet	94708	Folldal, Os, Tolga, Engerdal, Rendalen, Etnedal, Alvdal, Lom, Dovre, Stor-Elvdal, Skjåk, Våler, Vestre-Slidre, Lesja, Vang, Grue, Tynset, Nord-Odal, Vågå, Sør-Aurdal, Eidskog, Øystre-Slidre, Sør-Fron
Vestfold og Telemark	55170	Siljan, Fyresdal, Tokke, Hjartdal, Kviteseid, Nissedal, Seljord, Drangedal, Nome, Kragerø, Tinn
Agder	45661	Iveland, Bygland, Åmli, Hægebostad, Gjerstad, Valle, Vegårshei, Evje og Hornnes, Åseral, Birkenes, Sirdal, Bykle
Vestland	62604	Fedje, Modalen, Solund, Ulvik, Hyllestad, Eidfjord, Aurland, Samnanger, Masfjorden, Lærdal, Austrheim, Vik, Fitjar, Gulen, Fjaler, Askvoll, Etne, Vaksdal, Tysnes, Sveio, Høyanger
Trøndelag	51378	Leka, Røyrvik, Osen, Namsskogan, Høylandet, Rindal, Tydal, Flatanger, Lierne, Snåase-Snåsa, Holtålen, Frosta, Meråker, Rennebu, Selbu, Overhalla, Grong, Åfjord, Frøya
Troms og Finnmark	30140	Unjárga - Nesseby, Loppa, Loabák - Lavangen, Berlevåg, Kvænangen, Gratangen, Hasvik, Gamvik, Vardø, Dyrøy, Lebesby, Kárášjohka - Karasjok, Gáivuotna - Kåfjord - Kaivuono, Måsøy, Båtsfjord, Guovdageaidnu - Kautokeino, Salangen, Storfjord - Omasvuotna - Omasvuono, Ibestad

F. Multiple Intervention Linear Regression

The model building followed this pipeline: We first identified the different categories of interventions, by analysing the different COVID-19 interventions that had been utilised in Norway and qualitatively categorising interventions in groups, see details below. We chose to use a linear regression model to increase interpretability of the results. We did consider using raw mobility as the outcome rather than the log of the relative change in mobility from before and after the intervention, but if we pursued such a model, we would not have been able to use data from multiple regions. It was important that we could train a model using data from multiple regions, as we did not have enough data from any one region to create a separate model.

I. Model Details

$T_{I,r}$ represents the time points when an NPI was implemented in region r, where $r \in R$
$T_{C,r}$ represents the time points that are midpoints of two-week time periods when no NPI was implemented in region r, where $r \in R$
We denote the set of all time points for region r as $T_r = T_{I,r} \cup T_{C,r}$.
The models predict the log differences of the mobility after and before the intervention, computed using equation 2. We utilise daily mean meanDistAway, timeAway, and maxDistAway as the mean metrics.

$$Y_{r,t} = log(\sum_{p=t}^{t+6} MeanMetric_{r,p}) - log(\sum_{p=t-7}^{t-1} MeanMetric_{r,p}), r \in R, t \in T_r \quad (2)$$

For all models, the variance inflation factor was computed for each feature to identify potential multi-collinearities. Almost all variance inflation factor (VIF) values are under 5, which is below the recommended upper threshold [56]. Therefore, multicollinearity is not a significant issue.

For some intervention time points, the time period of comparison overlapped with either another intervention or a major holiday period, defined as the time around Easter, Christmas, and New Year's. We manually created new periods of analysis for this intervention and used equation 3 to compute the log differences in weekly mobility for these interventions. Table S11 lists the time points for which equation 3 was used to compute the outcome.

$$Y_{r,t} = log(\sum_{p=afterStart}^{afterEnd} MeanMetric_{r,p}) - log(\sum_{p=beforeStart}^{beforeEnd} MeanMetric_{r,p}), r \in R, t \in T_r \quad (3)$$

Table S10. Time Points with Modified Comparison Time Frames. For some interventions, time frames for the periods before and after the intervention of interest were modified to avoid overlap with holiday periods or other interventions. Equation 3 is used to compute the change in mobility for these intervention time points.

Time point	beforeStart	beforeEnd	afterStart	afterEnd
3-25	3-18	3-24	4-8	4-14
3-27	3-20	3-26	3-27	3-30
3-31	3-27	3-30	3-31	4-6
12-3	11-26	12-2	12-3	12-8
12-9	12-3	12-8	12-9	12-14
12-15	12-9	12-14	1-2	1-8

II. All Results from Models

We report all covariates and their coefficients in all three linear regression models.

Table S11. Results of Linear Regressions to Understand Effects of NPIs on Mobility Changes. The coefficients for all covariates representing different categories of NPIs are reported, along with a 95% confidence interval and corresponding p-value.

Intervention Category	meanDistAway		timeAway		maxDistAway	
	Coefficient (95% Confidence Interval)	P-Value	Coefficient (95% Confidence Interval)	P-Value	Coefficient (95% Confidence Interval)	P-Value
Intercept	0.00 (-0.021, 0.030)	0.707	0.00 (-0.01, 0.01)	0.884	0.01 (-0.01, 0.03)	0.343
StricterWH	0.02 (-0.07, 0.12)	0.615	-0.02 (-0.05, 0.02)	0.394	-0.01 (-0.09, 0.08)	0.892
WHEased	0.09 (-0.02, 0.20)	0.097	0.02 (-0.03, 0.06)	0.438	0.06 (-0.04, 0.16)	0.263
FMReq	-0.02 (-0.09, 0.06)	0.682	-0.01 (-0.04, 0.02)	0.350	0.01 (-0.06, 0.08)	0.816
FMRec	0.09 (-0.04, 0.22)	0.174	0.02 (-0.03, 0.07)	0.396	0.06 (-0.06, 0.17)	0.344
FMEased	0.01 (-0.06, 0.09)	0.717	0.00 (-0.02, 0.03)	0.741	0.02 (-0.05, 0.09)	0.627
StricterMeterRule	-0.17 (-0.34, -0.00)	0.047	-0.03 (-0.10, 0.03)	0.332	-0.14 (-0.29, 0.02)	0.084
LessSocDist	0.01 (-0.13, 0.16)	0.843	-0.06 (-0.11, -0.01)	0.030	0.00 (-0.13, 0.13)	0.984
PHDecr	-0.10 (-0.21, 0.02)	0.110	0.01 (-0.04, 0.05)	0.790	-0.06 (-0.17, 0.05)	0.258
PHIncr	0.02 (-0.06, 0.10)	0.581	0.02 (-0.01, 0.05)	0.206	0.06 (-0.02, 0.13)	0.129
LessAlc	0.09 (-0.01, 0.19)	0.084	-0.01 (-0.05, 0.03)	0.661	0.08 (-0.01, 0.17)	0.090
AddAlc	-0.04 (-0.14, 0.05)	0.371	0.03 (-0.01, 0.07)	0.092	-0.02 (-0.11, 0.07)	0.700
EventNumDecr	-0.06 (-0.19, 0.06)	0.303	0.01 (-0.03, 0.06)	0.527	-0.05 (-0.16, 0.06)	0.340
EventNumIncr	-0.01 (-0.08, 0.06)	0.832	0.00 (-0.03, 0.02)	0.870	-0.03 (-0.09, 0.03)	0.361

SchoolLimitsEased	0.06 (-0.05, 0.18)	0.249	0.00 (-0.05, 0.04)	0.871	0.03 (-0.07, 0.13)	0.573
StricterSchoolLimits	-0.02 (-0.23, 0.18)	0.826	0.00 (-0.15, 0.00)	0.063	0.00 (-0.19, 0.18)	0.961
GymsClosed	0.00 (0.00, 0.00)	0.205	0.00 (0.00, 0.00)	0.823	0.00 (0.00, 0.00)	0.338
GymsReopen	-0.09 (-0.27, 0.10)	0.349	-0.03 (-0.10, 0.04)	0.438	-0.18 (-0.35, -0.01)	0.037
ResShopReopen	0.08 (-0.02, 0.19)	0.117	0.07 (0.03, 0.11)	0.001	0.07 (-0.03, 0.16)	0.164
ResShopClose	-0.05 (-0.26, 0.16)	0.643	-0.01 (-0.09, 0.07)	0.858	-0.05 (-0.24, 0.15)	0.632

III. Intervention Categories to Intervention Dates

Table S12. Mapping of Intervention Categories to Dates of Implementation for each Region. Interventions are included both nationally and from Oslo, Bergen, Trondheim, Tromsø, and Stavanger. We do not include local December interventions around the 3rd, 9th, or 15th, as there are so many national interventions that were added at that time point.

Feature in Model	Corresponding Interventions	National	Oslo	Bergen	Trondheim	Tromsø	Stavanger
StricterWH	Work from home required, Normal office to Work from home recommended	12/15, 12/3, 12/9		2/7	2/10, 6/1	11/9, 10/28	4/16
WHEased	Work from home removed or becomes less mandatory	6/20	7/5	2/21, 5/31	6/22		5/6
FMReq	Face mask required	12/9, 12/15		2/7, 4/19, 8/12	11/24, 5/19, 8/26	11/9, 3/5	9/1
FMRec	Face mask recommended	3/25, 12/3	11/24	8/5	11/2	7/2, 10/28	
FMEased	Face mask measures eased			9/1	6/22, 9/21	3/19, 7/13, 11/30	9/15
StricterMeterRule	One metre rule added and recommendation to reduce close contacts	3/25, 12/3				10/28	
LessSocDist	Removing recommendation to social	9/25	6/16				

	distance or easing metre rule						
PHDecr	Private home limit decreased or made more mandatory		3/2	2/7, 4/19, 8/5, 8/12	6/1	3/5	
PHIncr	Private home limit increased or made less mandatory	2/3, 4/16, 6/20	6/16	5/12, 2/21, 5/31, 9/1	6/22	3/19	
LessAlc	Adding pouring stops or requirement of 1 metre at bars	3/25, 12/9, 12/15		2/7, 5/12	6/1, 2/10		
AddAlc	Easing pouring stops or 1 metre at pubs	4/16, 5/27, 6/20, 9/25	5/26, 6/16, 7/5	2/21, 5/31	2/2, 6/22		5/6
EventNumDecr	Number of people at events decreased, or event restrictions	3/25, 12/9, 12/15	3/2	2/7, 4/19	9/2	11/9	4/16
EventNumIncr	Number of people at events increased, or less event restrictions	2/3, 2/23, 4/16, 5/27, 6/20, 9/4, 9/25	5/26, 6/16	5/31, 2/21		11/30	5/6, 7/8
SchoolLimitsEased	less restrictions on schools	4/16	2/3, 4/19	2/21			
StricterSchoolLimits	more restrictions on schools		3/2	2/7, 4/19			
GymsClosed	gyms closed			3/27		3/5	
GymsReopen	gyms reopen			3/31		3/19	
ResShopReopen	Restaurants and businesses can reopen		2/3, 5/6, 5/26				
ResShopClose	Restaurants and shops closed		3/2		2/10		

IV. Creation of Control Time Points

The algorithm to identify non-intervention time points involves the following steps.

1. Start with a set of all dates, D. For each date t in the list of all national and local intervention dates, remove all dates from t-7 to t+6, inclusive. Only use national interventions when the region of interest is Norway.
2. Remove dates related to holidays. We removed one week before and after 12-28 and 4-5 to account for the winter and Easter holidays.

3. Iterate through dates in D in chronological order. For each date t, add t to the control time point list ($T_{C,r}$) if the t-7 and t+6 are still in D. Then, remove all dates from t-7 to t-6, inclusive from D.

V. Residual Plots

Figure S2. Residuals versus Predicted Values for Linear Regressions. This analysis shows that there are no outliers and relatively consistent variance.

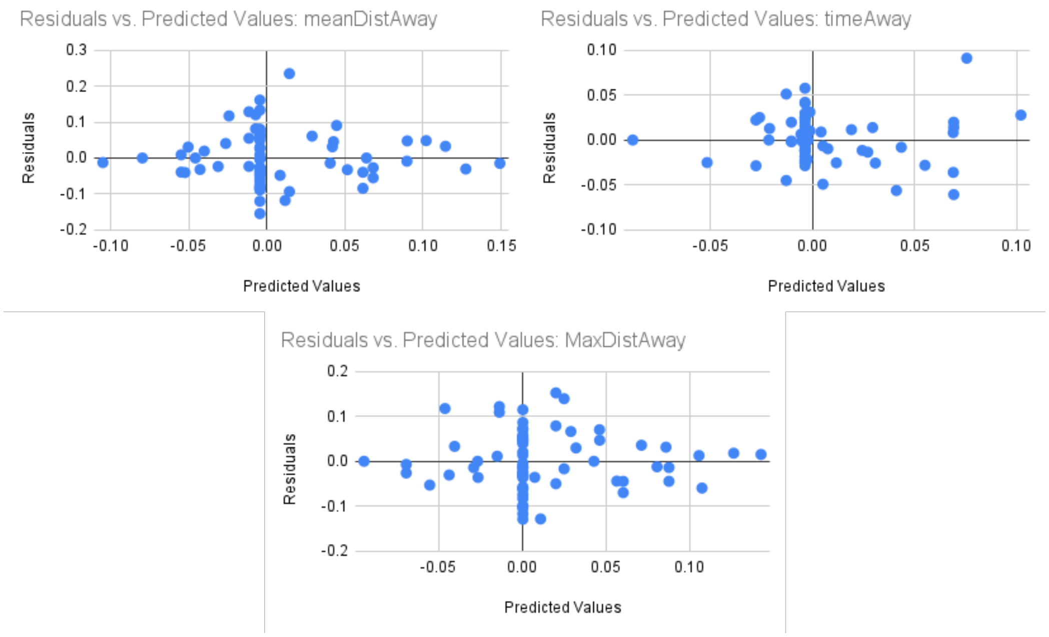

References

1. Ministry of Health and Care Services. Infection control measures are continued – but restrictions eased for children and young people [Internet]. regjeringen.no. 2021 [cited 2022Mar2]. Available from: https://www.regjeringen.no/en/historical-archive/solbergs-government/Ministries/hod/News/2021ny/infection-control-measures-are-continued-but-restrictions-eased-for-children-and-young-people/id2830766/
2. Ministry of Health and Care Services. The government is implementing stricter national measures [Internet]. regjeringen.no. 2021 [cited 2022Mar2]. Available from: https://www.regjeringen.no/en/historical-archive/solbergs-government/Ministries/hod/News/2021ny/the-government-is-implementing-stricter-national-measures/id2841039/
3. Statsministerens kontor. Regjeringen starter på første trinn I Gjenåpningsplanen [Internet]. regjeringen.no. [cited 2022Mar2]. Available from: https://www.regjeringen.no/no/dokumentarkiv/regjeringen-solberg/aktuelt-regjeringen-solberg/smk/pressemeldinger/2021/regjeringen-starter-pa-forste-trinn-i-gjenapningsplanen/id2844380/
4. Klesty V, Adomaitis N. Norway to ease covid-19 restrictions further next week [Internet]. Reuters. Thomson Reuters; 2021 [cited 2022Mar2]. Available from: https://www.reuters.com/world/norway-further-ease-covid-19-restrictions-may-27-pm-says-2021-05-21/
5. The Office of the Prime Minister. The Norwegian government is continuing to reopen society [Internet]. regjeringen.no. 2021 [cited 2022Mar2]. Available from: https://www.regjeringen.no/en/historical-archive/solbergs-government/Ministries/smk/Press-releases/2021/the-norwegian-government-is-continuing-to-reopen-society/id2862266/

6. Endringslogg [Internet]. Helsedirektoratet. [cited 2022Mar2]. Available from: https://www.helsedirektoratet.no/veiledere/koronavirus/endringslogg
7. The Office of the Prime Minister. Norway moves to normal everyday life with increased emergency preparedness [Internet]. regjeringen.no. 2021 [cited 2022Mar2]. Available from: https://www.regjeringen.no/en/historical-archive/solbergs-government/Ministries/smk/Press-releases/2021/norway-moves-to-normal-everyday-life-with-increased-emergency-preparedness/id2872539/
8. The Office of the Prime Minister. The Norwegian government is introducing new national measures [Internet]. regjeringen.no. 2021 [cited 2022Mar2]. Available from: https://www.regjeringen.no/en/historical-archive/stoeres-government/utdaterte-aktueltsaker/smk/the-norwegian-government-is-introducing-new-national-measures/id2890578/
9. The Office of the Prime Minister. New national measures to keep the pandemic under control [Internet]. regjeringen.no. 2021 [cited 2022Mar2]. Available from: https://www.regjeringen.no/en/historical-archive/stoeres-government/utdaterte-aktueltsaker/smk/new-national-measures-to-keep-the-pandemic-under-control/id2891395/
10. The Office of the Prime Minister. Stricter national measures to limit the spread of Omicron [Internet]. regjeringen.no. [cited 2022Mar2]. Available from: https://www.regjeringen.no/en/historical-archive/stoeres-government/utdaterte-aktueltsaker/smk/stricter-national-measures-to-limit-the-spread-of-omicron/id2892042/
11. 1 February: The city government eases Corona measures in Oslo [Internet]. Oslo kommune. 2021 [cited 2022Mar2]. Available from: https://www.oslo.kommune.no/english/coronavirus/status-reports-on-coronavirus-measures/1-february-the-city-government-eases-corona-measures-in-oslo#gref
12. Mordt H, Silseth I, Sandøy CI, Malm M. Pressekonferanse om Nye Strengere koronatiltak etter vinterferien [Internet]. NRK. NRK; 2021 [cited 2022Mar2]. Available from: https://www.nrk.no/osloogviken/pressekonferanse-om-nye-strengere-koronatiltak-etter-vinterferien-1.15395047
13. 14 April: Oslo city government introduces enhanced red risk level at schools in Oslo – students can return to class [Internet]. Oslo kommune. 2021 [cited 2022Mar2]. Available from: https://www.oslo.kommune.no/english/coronavirus/status-reports-on-coronavirus-measures/14-april-oslo-city-government-introduces-enhanced-red-risk-level-at-schools-in-oslo-students-can-return-to-class
14. 5 May: Oslo city government continues its gradual reopening of Oslo [Internet]. Oslo kommune. 2021 [cited 2022Mar2]. Available from: https://www.oslo.kommune.no/english/coronavirus/status-reports-on-coronavirus-measures/5-may-oslo-city-government-continues-its-gradual-reopening-of-oslo#gref
15. 21 May: Oslo city government continues its phased reopening of Oslo [Internet]. Oslo kommune. 2021 [cited 2022Mar2]. Available from: https://www.oslo.kommune.no/english/coronavirus/status-reports-on-coronavirus-measures/21-may-oslo-city-government-continues-its-phased-reopening-of-oslo#gref
16. 15 June: Oslo City Government continues to reopen Oslo [Internet]. Oslo kommune. 2021 [cited 2022Mar2]. Available from: https://www.oslo.kommune.no/english/coronavirus/status-reports-on-coronavirus-measures/15-june-oslo-city-government-continues-to-reopen-oslo
17. 1 July: Oslo continues to reopen [Internet]. Oslo kommune. 2021 [cited 2022Mar2]. Available from: https://www.oslo.kommune.no/english/coronavirus/status-reports-on-coronavirus-measures/1-july-oslo-continues-to-reopen#gref
18. 24 November: New coronavirus (covid-19) guidance and enhanced measures for the city of Oslo [Internet]. Oslo kommune. 2021 [cited 2022Mar2]. Available from: https://www.oslo.kommune.no/english/coronavirus/status-reports-on-coronavirus-measures/24-november-new-coronavirus-covid-19-guidance-and-enhanced-measures-for-the-city-of-oslo#gref
19. 13 Desember: Red and yellow levels for kindergartens and schools in Oslo [Internet]. Oslo kommune. 2021 [cited 2022Mar2]. Available from: https://www.oslo.kommune.no/english/coronavirus/status-reports-on-coronavirus-measures/13-desember-red-and-yellow-levels-for-kindergartens-and-schools-in-oslo#gref

20. Solheim P, Røyne H, Hagfors CL, Røset HH. Innfører ringsystem for Bergensområdet [Internet]. VG. 2021 [cited 2022Mar2]. Available from: https://www.vg.no/nyheter/innenriks/i/Alpzaz/innfoerer-ringsystem-for-bergensomraadet
21. Anbefaler at Endring av Tiltaksnivå Skal Gjelde fra og med mandag [Internet]. Bergen kommune. 2021 [cited 2022Mar2]. Available from: https://www.bergen.kommune.no/hvaskjer/tema/presserom/pressemeldinger/anbefaler-at-endring-av-tiltaksniva-skal-gjelde-fra-og-med-mandag
22. Lyngstad HK, Bugge S. Smitteutbrudd i Bergen: Stenger alle Treningssentre [Internet]. VG. 2021 [cited 2022Mar2]. Available from: https://www.vg.no/nyheter/innenriks/i/we29z5/smitteutbrudd-i-bergen-stenger-alle-treningssentre
23. Treningssentre Kan åpne med strenge restriksjoner [Internet]. Bergen kommune. 2021 [cited 2022Mar2]. Available from: https://www.bergen.kommune.no/hvaskjer/tema/koronavirus/siste-nytt/treningssentre-kan-apne-med-strenge-restriksjoner
24. Valhammer BR. Bergen Varsler Nye Coronatiltak Klokken 14 – VG NÅ: Coronaviruset [Internet]. VG. 2021 [cited 2022Mar2]. Available from: https://direkte.vg.no/coronaviruset/news/bergen-varsler-nye-coronatiltak-klokken-14.I6luyorqh
25. Sviggum SK. Bergen: Letter på besøksreglene, Innstrammer for utelivet – VG NÅ: Coronaviruset [Internet]. VG. 2021 [cited 2022Mar2]. Available from: https://direkte.vg.no/coronaviruset/news/bergen-strammer-inn-smittevernsreglene.yR4eVpbUf
26. Røyne H. Bergen letter på smitteverntiltakene – VG NÅ: Coronaviruset [Internet]. VG. 2021 [cited 2022Mar2]. Available from: https://direkte.vg.no/coronaviruset/news/bergen-letter-paa-smitteverntiltakene.EQ54GD_vv
27. Solberg K. Smitteutbruddet i Bergen: Innfører Coronatiltak – VG NÅ: Coronaviruset [Internet]. VG. 2021 [cited 2022Mar2]. Available from: https://direkte.vg.no/coronaviruset/news/smitteutbruddet-i-bergen-innfoerer-coronatiltak.qfMrB4rzU
28. Hægeland L, Ording O, Hagfors CL. Nye Coronatiltak i Bergen: – Vi ønsker Alle Å Bli Ferdig Med Denne Pandemien [Internet]. VG. 2021 [cited 2022Mar2]. Available from: https://www.vg.no/nyheter/innenriks/i/OryWk1/nye-coronatiltak-i-bergen-vi-oensker-alle-aa-bli-ferdig-med-denne-pandemien
29. Solberg K. Bergen går bort fra coronaforskrift – VG NÅ: Coronaviruset [Internet]. VG. 2021 [cited 2022Mar2]. Available from: https://direkte.vg.no/coronaviruset/news/bergen-gaar-bort-fra-coronaforskrift.AYDy-m-Ni
30. Nave OB. Bergen: Utvider Munnbindpåbud – VG NÅ: Coronaviruset [Internet]. VG. 2021 [cited 2022Mar2]. Available from: https://direkte.vg.no/coronaviruset/news/bergen-utvider-munnbindpaabud.Wzl8vBnq8
31. Disse innfører digital skole – VG NÅ: Coronaviruset [Internet]. VG. 2021 [cited 2022Mar2]. Available from: https://direkte.vg.no/coronaviruset/news/oslo-innfoerer-digital-skole.K_NPNbLQP
32. Røyne H. Trondheim Tillater alkoholservering Fram til midnatt – vg nå: Coronaviruset [Internet]. VG. 2021 [cited 2022Mar2]. Available from: https://direkte.vg.no/coronaviruset/news/trondheim-tillater-alkoholservering-fram-til-midnatt.3rs0oGg51
33. Hellstrand SH. Vil innføre Skjenkestopp I trondheim – VG NÅ: Coronaviruset [Internet]. VG. 2021 [cited 2022Mar2]. Available from: https://direkte.vg.no/coronaviruset/news/vil-innfoere-skjenkestopp-i-trondheim.ih6gVyZFJ
34. Lode SC. Dette er de Nye tiltakene I Trondheim – VG NÅ: Coronaviruset [Internet]. VG. 2021 [cited 2022Mar2]. Available from: https://direkte.vg.no/coronaviruset/news/dette-er-de-nye-tiltakene-i-trondheim.5f5rD6Si0
35. NÅ Stenger Trondheim Ned: - det er villsmitte og indisk mutant på ferde i Byen Vår – VG NÅ: Coronaviruset [Internet]. VG. 2021 [cited 2022Mar2]. Available from:

https://direkte.vg.no/coronaviruset/news/naa-stenger-trondheim-ned-det-er-villsmitte-og-indisk-mutant-paa-ferde-i-byen-vaar.ggZ6-tFCV
36. Solberg K. Trondheim åpner Opp – VG NÅ: Coronaviruset [Internet]. VG. 2021 [cited 2022Mar2]. Available from: https://direkte.vg.no/coronaviruset/news/trondheim-aapner-opp.evba6RI_2
37. Solberg K. Trondheim innfører påbud om munnbind – VG NÅ: Coronaviruset [Internet]. VG. 2021 [cited 2022Mar2]. Available from: https://direkte.vg.no/coronaviruset/news/tv-2-trondheim-innfoerer-paabud-om-munnbind.T8X1wfQFJ
38. Anbefaler studentene Å avlyse Eller Utsette store arrangement [Internet]. Trondheim kommune. 2021 [cited 2022Mar2]. Available from: https://www.trondheim.kommune.no/aktuelt/nyhetssaker/korona/2021/august/anbefaler-studentene-a-avlyse-eller-utsette-store-arrangement/
39. Nave OB. Trondheim opphever munnbind-påbud – VG NÅ: Coronaviruset [Internet]. VG. 2021 [cited 2022Mar2]. Available from: https://direkte.vg.no/coronaviruset/news/trondheim-opphever-munnbind-paabud.A4Z7luCEs
40. Skjetne OL, Bugge S, Bjaarstad JY-E, Lode SC. Trondheim Kommune Anbefaler munnbind igjen [Internet]. VG. 2021 [cited 2022Mar2]. Available from: https://www.vg.no/nyheter/innenriks/i/g641y9/trondheim-kommune-anbefaler-munnbind-igjen
41. Trondheim innfører påbud om munnbind – VG NÅ: Coronaviruset [Internet]. VG. 2021 [cited 2022Mar2]. Available from: https://direkte.vg.no/coronaviruset/news/trondheim-innfoerer-paabud-om-munnbind.ieV805ucg
42. Olsen DL. Nye tiltak I Tromsø – VG NÅ: Coronaviruset [Internet]. VG. 2021 [cited 2022Mar2]. Available from: https://direkte.vg.no/coronaviruset/news/ny-tiltak-i-tromsoe.e13ZWprMk
43. Machlar SS. Munnbindanbefalinger i Tromsø – VG NÅ: Coronaviruset [Internet]. VG. 2021 [cited 2022Mar2]. Available from: https://direkte.vg.no/coronaviruset/news/munnbindanbefalinger-i-tromsoe.QxKiTtcjo
44. Nyheter og tall [Internet]. Tromsø kommune. [cited 2022Mar2]. Available from: https://tromso.kommune.no/korona/nyheter-og-tall
45. Sviggum SK. Tromsø kommune gjeninnfører Coronameteren Etter smitteøkning – VG NÅ: Coronaviruset [Internet]. VG. 2021 [cited 2022Mar2]. Available from: https://direkte.vg.no/coronaviruset/news/tromsoe-kommune-gjeninnfoerer-coronameteren.qvYAHQTZt
46. Oppdaterte Smittevernregler for tromsø kommune [Internet]. Tromsø kommune. 2021 [cited 2022Mar2]. Available from: https://tromso.kommune.no/nyheter/2021/11/oppdaterte-smittevernregler-tromso-kommune
47. Ording O. Tromsø Lemper på smitteverntiltak – VG NÅ: Coronaviruset [Internet]. VG. 2021 [cited 2022Mar2]. Available from: https://direkte.vg.no/coronaviruset/news/tromsoe-lemper-paa-smitteverntiltak.F3gpBle8y
48. Nave OB. Nord-Jæren Innfører strengere tiltak – VG NÅ: Coronaviruset [Internet]. VG. 2021 [cited 2022Mar2]. Available from: https://direkte.vg.no/coronaviruset/news/nord-jaeren-innfoerer-strengere-tiltak.bRyewwxsZ
49. Kommunene på nord-Jæren går over til nasjonalt tiltaksnivå [Internet]. Stavanger kommune. 2021 [cited 2022Mar2]. Available from: https://www.stavanger.kommune.no/nyheter/ordforerne-i-kommunene-stavanger-randaberg-sola-og-sandnes-har-vedtatt-a-ga-over-til-nasjonalt-tiltaksniva/
50. Nave OB. Lokale Tiltak på nord-Jæren forlenges I én uke – VG NÅ: Coronaviruset [Internet]. VG. 2021 [cited 2022Mar2]. Available from: https://direkte.vg.no/coronaviruset/news/nord-jaeren-forlenger-lokale-smitteverntiltak-i-en-uke-til.OZPjduwMz
51. Innfører Nytt munnbind-påbud I Kollektivtrafikken og Drosjer på nord-Jæren [Internet]. Stavanger kommune. 2021 [cited 2022Mar2]. Available from: https://www.stavanger.kommune.no/nyheter/innforer-nytt-munnbind-pabud-i-kollektivtrafikken-og-drosjer-pa-nord-jaren/

52. Innfører munnbind-påbud PÅ nord-jæren [Internet]. Stavanger kommune. 2021 [cited 2022Mar2]. Available from: https://www.stavanger.kommune.no/nyheter/innforer-munnbind-pabud-pa-nord-jaren/
53. Storaas IH. Gult Nivå på skolene I Stavanger – VG NÅ: Coronaviruset [Internet]. VG. 2021 [cited 2022Mar2]. Available from: https://direkte.vg.no/coronaviruset/news/gult-nivaa-paa-skolene-i-stavanger.-CJmN7NEo
54. Haugen V, Nave OB, Solberg K. Disse Innfører Digital skole [Internet]. VG. 2021 [cited 2022Mar2]. Available from: https://www.vg.no/nyheter/innenriks/i/pW4ApV/disse-innfoerer-digital-skole
55. James G, Witten D, Hastie T, Tibshirani R. An Introduction to Statistical Learning: With Applications in R. 1st ed. 2013, Corr. 7th printing 2017 edition. Springer; 2013.
